# Obsessive-compulsive symptoms in professional tennis players

**DOI:** 10.1192/j.eurpsy.2024.732

**Published:** 2024-08-27

**Authors:** R. Gurrieri, A. Arone, E. Parra, S. Palermo, D. Marazziti, A. Gemignani

**Affiliations:** ^1^Department of Surgical- Medical and Molecular Pathology and Critical Care Medicine; ^2^Department of Clinical and Experimental Medicine, University of Pisa, Pisa; ^3^Saint Camillus International University of Health and Medical Sciences, Unicamillus, Roma, Italy

## Abstract

**Introduction:**

Engaging in moderate physical activity holds a vital role in our daily lives, serving as both a means of social recreation and a fundamental contributor to physical and mental wellbeing. It is also worth noting that such activity can potentially produce mood-enhancing effects by promoting neurogenesis and neuronal adaptability. Intriguingly, certain individual psychological traits such as rituals, compulsions, obsessional thinking, and superstitious beliefs, as well as inflexibility in daily routines, appear to serve a purpose in competitive athletic endeavors.

**Objectives:**

The aim of our study was to investigate the possible presence of obsessive-compulsive symptoms or disorders, as well as of superstitions or magical thinking, in a group of professional tennis players, by means of standardized assessment scales, as compared with healthy subjects who did not professionally perform any kind of sport activity.

**Methods:**

Twenty-five current or former professional tennis were recruited within the Italian Tennis Federation during an international competition and during a master meeting of coaches. All of them underwent a psychiatric interview with a structured scale and a psychopathological assessment carried out with the Mini-International Neuropsychiatric Interview (MINI) and the Yale-Brown Obsessive-Compulsive Scale (Y-BOCS). Data were analyzed and compared.analysis was performed by means of contingency tables, χ² tests, group statistics, paired, independent and Mann Whitney’s tests.

**Results:**

The Y-BOCS total score was significantly higher in both current and retired athletes than control subjects (5.96 ± 5.76 versus 1.24 ± 2.65, p = 0.001, t = 3.72). Current athletes showed more frequently current aggressive obsessions (χ2 = 0.041, r = 5.24) and current miscellaneous compulsions (χ2 = 0.030, r = 5.94) than past athletes. The Y-BOCS (t = 3.4, p = 0.002) obsessions (t = 3.48, p = 0.002), and compulsions subscale (t = 3.11, p = 0.005) scores were higher in current players than in the other group.

**Conclusions:**

Our results support the hypothesis that high-level competitive sports activities, which suppose compliance with strict daily routines and extensive training, could constitute a risk factor for the onset of full-blown obsessive-compulsive disorder in more vulnerable subjects. Similarly, there is a growing demand for sport psychological support experts in order to prevent high stress in training and competitions.

**Disclosure of Interest:**

None Declared

